# The *you* Gene Encodes an EGF-CUB Protein Essential for Hedgehog Signaling in Zebrafish

**DOI:** 10.1371/journal.pbio.0030066

**Published:** 2005-01-11

**Authors:** Ian G Woods, William S Talbot

**Affiliations:** **1**Department of Developmental Biology, Stanford University School of MedicineStanford, CaliforniaUnited States of America; Wellcome Trust Sanger InstituteUnited Kingdom

## Abstract

Hedgehog signaling is required for many aspects of development in vertebrates and invertebrates. Misregulation of the Hedgehog pathway causes developmental abnormalities and has been implicated in certain types of cancer. Large-scale genetic screens in zebrafish have identified a group of mutations, termed *you-*class mutations, that share common defects in somite shape and in most cases disrupt Hedgehog signaling. These mutant embryos exhibit U-shaped somites characteristic of defects in slow muscle development. In addition, Hedgehog pathway mutations disrupt spinal cord patterning. We report the positional cloning of *you,* one of the original *you*-class mutations, and show that it is required for Hedgehog signaling in the development of slow muscle and in the specification of ventral fates in the spinal cord. The *you* gene encodes a novel protein with conserved EGF and CUB domains and a secretory pathway signal sequence. Epistasis experiments support an extracellular role for You upstream of the Hedgehog response mechanism. Analysis of chimeras indicates that *you* mutant cells can appropriately respond to Hedgehog signaling in a wild-type environment. Additional chimera analysis indicates that wild-type *you* gene function is not required in axial Hedgehog-producing cells, suggesting that You is essential for transport or stability of Hedgehog signals in the extracellular environment. Our positional cloning and functional studies demonstrate that You is a novel extracellular component of the Hedgehog pathway in vertebrates.

## Abstract 2

Genetic studies in zebrafish have identified a new protein involved in Hedgehog signaling - a key pathway required for development in vertebrates and invertebrates

## Introduction

The coordination of growth, proliferation, and differentiation during development requires transmission of information in the form of extracellular signals. Hedgehog signaling is of fundamental importance in the development of a wide variety of tissues and organ systems. Much of the initial functional analysis of Hedgehog signaling focused on the patterning of Drosophila larval segments and imaginal discs, dorsoventral patterning of the vertebrate neural tube, and anterior–posterior patterning of vertebrate limbs; in addition, many recent studies have illuminated the widespread and conserved role of Hedgehog signaling in development (reviewed in [1]). Misregulation of Hedgehog signaling has been implicated in several diseases and developmental abnormalities, including basal cell carcinoma [2,3,4], medulloblastoma [5,6,7], pancreatic cancer [8], and holoprosencephaly [9,10].

After release from signaling cells, the activity and distribution of Hedgehog proteins are modulated by a variety of factors in the extracellular environment. In Drosophila, diffusion of lipid-modified Hedgehog proteins is dependent on the action of *tout velu,* a gene involved in the synthesis of heparan sulfate proteoglycans [11,12]. The diffusion of Hedgehog is also attenuated via sequestration by its receptor, Patched [13]. In vertebrates, Hedgehog proteins may be further regulated by binding to the *growth-arrest specific* gene product Gas1 [14], and Hedgehog-interacting protein Hip1, which is itself induced by Hedgehog signaling [15]. Moreover, the ability of Hedgehog proteins to diffuse over significant distances in the developing vertebrate limb bud appears to depend on the cholesterol modification of the Hedgehog protein; this modification may facilitate the assembly of Hedgehog proteins into a multimeric structure, perhaps conferring increased stability or mobility [16,17]. Genetic and biochemical evidence suggests that the low-density receptor-related protein Megalin may also play a role in Hedgehog signaling in vertebrates, perhaps by binding to Hedgehog proteins and facilitating their endocytosis [18,19].

Hedgehog pathway function in zebrafish has been analyzed primarily in the context of skeletal muscle development and differentiation [20,21,22,23,24,25,26,27,28]. In zebrafish embryos at 24 h post fertilization (hpf), skeletal muscle can be subdivided into two distinct classes based on morphological characteristics and gene expression. Slow muscle fibers are mononucleate, express characteristic slow muscle forms of myosin heavy chain, and show strong nuclear expression of the transcription factor *prox1*. In contrast, fast muscle fibers can be identified via their multinucleate morphology and lack of *prox1* expression [28]. Cell labeling experiments have demonstrated that slow muscle fibers derive from the adaxial cells that lie immediately adjacent to the notochord [21]. As development progresses, a subset of these developing slow muscle cells migrates laterally through the myotome to form the superficial slow fibers [21,27,28]. Slow muscle fibers that remain near the midline—the muscle pioneers—express high levels of Engrailed and organize the somites into their distinctive chevron shape [22,28,29,30]. The remaining muscle cells in the interior of the myotome form multinucleate fast muscle fibers [21,28].

Many lines of evidence indicate that Hedgehog signals from axial tissues specify slow muscle in zebrafish. Slow muscle fibers are reduced or absent in embryos with Hedgehog pathway mutations [25,26,31,32,33]. Conversely, slow muscle is expanded at the expense of fast muscle in embryos with increased Hedgehog pathway activity [20,23,24,34]. Moreover, addition of Hedgehog protein to cultured zebrafish myoblasts induces expression of slow-muscle-specific forms of myosin heavy chain [35].

Genetic screens have identified a number of mutations disrupting the Hedgehog pathway in zebrafish [32,33,36,37,38,39,40,41]. Many of these Hedgehog pathway mutants share characteristic defects, the most obvious of which is abnormal somite morphology resulting from disrupted slow muscle specification and the lack of horizontal myoseptum [22]. These mutants are thus termed “*you*-class” mutants because of their U-shaped somites. Five of the seven *you*-class mutations have been cloned, and four of these genes, *syu/shh, yot/gli2, smu/smoh,* and *con/disp1,* encode members of the Hedgehog signaling pathway [32,33,36,37,39]. The exception is *ubo/prdm1,* which encodes a transcriptional switch that acts downstream of Hedgehog signaling in the development of slow muscle [27,42]. Careful analysis reveals differences between the *ubo* and Hedgehog pathway mutant phenotypes. For example, Hedgehog pathway mutants have defects in the lateral floor plate of the neural tube and the dorsal aorta, which are apparently normal in *ubo* mutants [22,43]. Examination of Hedgehog-induced gene expression also reveals a clear distinction between Hedgehog pathway mutations and *ubo:* Hedgehog pathway mutations reduce or abolish expression of the Hedgehog target *ptc1,* whereas *ptc1* expression is normal in *ubo* mutants, indicating that they can receive Hedgehog signals [22,25,26,32,33,36,39].

Previous phenotypic characterization of mutants for the eponymous *you*-class gene, *you,* has revealed delayed development of the dorsal aorta and the absence of lateral floor plate marker expression in addition to slow muscle defects [22,43]. Moreover, expression of Hedgehog target genes, including *ptc1* and adaxial *myod,* is reduced in *you* mutants [25]. These results suggest that the *you* gene acts within the Hedgehog pathway itself rather than downstream of Hedgehog signaling in processes specific to slow muscle development. Prior to this study, the molecular identity of the *you* gene has remained unknown. We report the positional cloning of the *you* gene and show that it encodes a novel extracellular EGF-CUB (epidermal growth factor–complement Uegf Bmp1) protein required for Hedgehog signaling. Functional studies provide evidence that *you* is essential for the transport or stability of Hedgehog signals in the extracellular environment.

## Results

### *you* Function Is Required for Hedgehog Signaling

In wild-type zebrafish embryos, the dorsal and ventral portions of each myotome converge at a point where the horizontal myoseptum forms, giving the somites their characteristic chevron shape (Figure 1A). In contrast, *you* mutants lacked the horizontal myoseptum and exhibit the U-shaped morphology that defines mutants of the *you* class (Figure 1B; [22]). The formation of the horizontal myoseptum in zebrafish depends on the proper development of slow muscle, a process that is defective in *you-*class mutants [22,31]. Hedgehog activity in the context of slow muscle cell specification can be assayed by analyzing Engrailed expression in the muscle pioneers and adaxial *myod* expression during somitogenesis. Wild-type embryos at 24 hpf exhibit strong Engrailed staining in characteristic elongate nuclei of 2–6 muscle pioneers—slow muscle cells that develop along the prospective horizontal myoseptum—per myotomal segment (Figure 1C). In addition, weaker Engrailed expression can be observed in the typically more rounded nuclei of mulitnucleate fast muscle fibers, which are situated farther from the horizontal myoseptum (Figure 1C; [28,30]). *you* mutant embryos completely lacked the strong Engrailed expression in the muscle pioneers (Figure 1D; [22]), but very weak labeling was sometimes observed in a small number of cells. When weakly expressing cells were present, they were confined to the nine most anterior somites. In wild-type embryos, *myod* is expressed during somitogenesis in the adaxial cells of both somitic and presomitic mesoderm, and laterally along the posterior borders of developing somites (Figure 1E). *you* mutant embryos retained the lateral expression of *myod,* but the adaxial expression of *myod* was absent in the trunk and reduced in the tail bud (Figure 1F; [25]). In addition, expression of *ptc1—*both a member of the Hedgehog pathway and a sensitive transcriptional readout of Hedgehog signaling—was reduced in the adaxial cells of *you* mutants at 22 somites and other stages (Figure 1G and 1H; data not shown; [25]).

**Figure 1:**
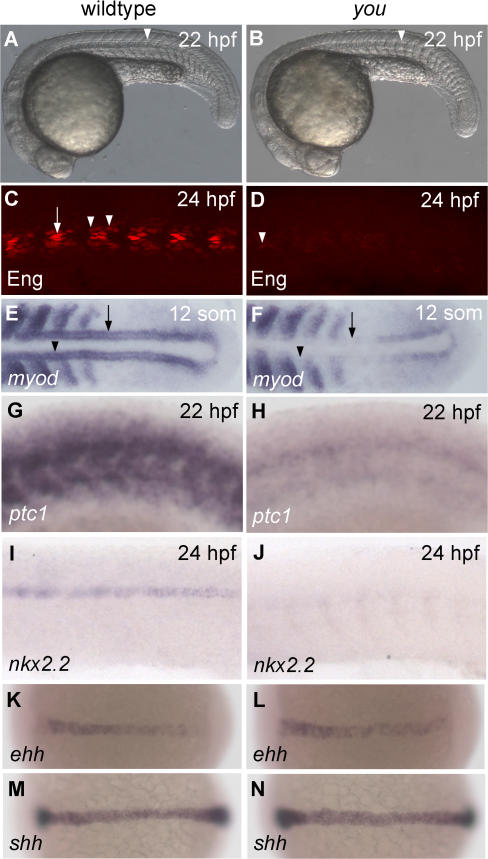
*you* Mutants Exhibit Hedgehog-Associated Defects in Slow Muscle and Ventral Spinal Cord (A and B) Lateral views of live zebrafish at 22 hpf. Wild-type embryos (A) have chevron-shaped somites and a clearly visible floor plate (arrowhead), while *you* mutants (B) exhibit U-shaped somites and an indistinct floor plate (arrowhead). (C and D) Lateral views of somites 8–13 in whole-mount embryos at 24 hpf. Wild-type embryos (C) show strong Engrailed expression in muscle pioneers (arrow), and weaker expression in multinucleate medial fast fibers (arrowheads). Engrailed expression in *you* mutants (D) is mostly absent, though very weak expression can occasionally be observed (arrowhead). (E and F) Dorsal views of posterior trunk and tail bud in 12-somite embryos. Wild-type embryos (E) exhibit adaxial *myod* expression throughout the somitic (arrowhead) and presomitic (arrow) mesoderm, while *you* mutants (F) lack expression in the somitic (arrowhead) and in parts of the presomitic (arrow) mesoderm. (G and H) Lateral view of somites 9–15 in whole-mount embryos at 22 hpf. Wild-type embryos (G) exhibit strong expression of *ptc1,* while *you* mutants (H) show weaker levels of *ptc1* expression. (I and J) Lateral view of spinal cord in the posterior trunk of whole-mount embryos at 24 hpf. Wild-type embryos (I) show expression of *nkx2.2* in the ventral spinal cord, while in *you* mutants (J) this expression is strongly reduced. (K–N) Dorsal views of whole-mount embryos at bud stage (10 hpf). Expression in *you* mutant embryos of both *ehh* (L) and *shh* (N) is similar to that observed wild-type embryos (K and M). Anterior is to the left in all images. Genotypes of all embryos were determined by PCR after photography.

In addition to these disruptions in developing somites, *you* mutant embryos showed defects in patterning of the central nervous system. *nkx2.2,* a Hedgehog-induced marker of ventral cell types in the spinal cord [44], was absent in the trunk and tail of *you* mutants (Figure 1I and 1J). Moreover, our analysis and prior work has revealed that *you* embryos show delayed and weakened blood circulation in the dorsal aorta (data not shown; [45]). These results and previous phenotypic analyses support the conclusion that *you* gene function is required for Hedgehog signaling in development of slow muscle, ventral spinal cord, and the dorsal aorta.

In zebrafish, three *hedgehog* genes*—ehh, shh, and twhh*—are expressed at the midline in early embryonic stages. To determine whether *you* is required for *hedgehog* gene transcription, we analyzed the expression of *hedgehog* genes in wild-type and *you* mutant embryos. Expression of all three *hedgehog* genes appeared normal in *you* embryos at bud stage (10 hpf; Figure 1K–1N; data not shown).

### Positional Cloning of *you*

As the first step toward identifying the *you* gene, we mapped the *you* mutation to a 1-cM (12 recombinants among 1,156 meioses) region of LG 7, between simple sequence length polymorphism (SSLP) markers Z11119 and Z15270 (Figure 2A). By comparing the position of the *you* mutation to zebrafish genetic maps ([46,47]; unpublished data), we excluded as candidate genes more than 60 zebrafish orthologs of Hedgehog pathway genes and genes known to interact with the Hedgehog pathway. We therefore adopted a positional cloning strategy to identify the gene disrupted by the *you* mutation. Using a marker linked with Z15270 on a contig from the Sanger Institute whole-genome shotgun assembly, we screened a pooled bacterial artificial chromosome (BAC) library and initiated a chromosome walk beginning with BAC zC172H20. After identifying polymorphisms in BAC end sequences, testing these polymorphisms on our mapping panel, and iteratively rescreening the pooled BAC library, we identified a contiguous stretch of genomic sequence spanning portions of five BACs with ends that flanked *you* (Figure 2A).

**Figure 2:**
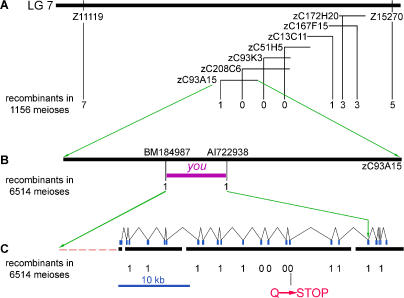
Positional Cloning of the *you* Locus (A) Genetic and physical map of the *you* region on LG 7, showing the initial flanking SSLPs and the BACs used in the chromosome walk. The number of recombinants in 1,156 meioses is shown for the SSLP markers and for mapped BAC end sequences. (B) Diagram of BAC zC93A15, with expressed sequence tag markers BM184987 and AI722938 shown flanking the *you* locus. Both BM184987 and AI722938 showed one recombinant out of 6,514 meioses, and were genetically localized on opposite sides of *you*. (C) The genomic region of the *you* locus is depicted by horizontal black bars. Gaps in these bars represent fragments of genomic sequence that were not obtained in the sequencing analysis. Exons are depicted by blue rectangles, and the number of recombinants in 6,514 meioses is shown below each mapped exon. Single nucleotide polymorphisms in four exons always segregated with the *you* locus, and one of these exons harbored a single nucleotide lesion predicted to change a glutamine codon to a stop codon and truncate the open reading frame.

To reduce the critical interval that contained *you,* we improved the resolution of our map by increasing our mapping panel to 6,514 meioses. By scoring sequences identified from BAC zC93A15 in key recombinants from the mapping panel, we localized the *you* gene to a portion of this BAC between polymorphisms identified in expressed sequence tag markers BM184987 and AI722938 (Figure 2B). Further sequence analysis and mapping identified other exons of the same gene as AI722938, some of which were on the opposite side of the mutation from the original AI722938 marker (Figure 2C). We isolated a full-length cDNA clone for this gene and used this new sequence information to identify polymorphisms in other exons. In all, high-resolution mapping identified four exons that failed to recombine with *you* and confirmed that the two ends of the gene flanked the *you^ty97^* mutation. Sequence analysis of the four non-recombining exons in wild-type and *you^ty97^* mutant genomic DNA identified a nonsense mutation that truncates the predicted protein approximately two-thirds of the way through the open reading frame. These findings, together with others described below, indicate that this gene is disrupted by the *you* mutation.

### *you* Is Orthologous to *Scube2*

The protein encoded by *you* comprises 1,010 amino acids, and comparison of the predicted *you* amino acid sequence against the protein database indicated that the *you* protein is highly similar to a family of proteins founded by mouse SCUBE1 (Signal sequence, CUB domain, EGF-related; [48]). Pairwise sequence comparisons between *you* and SCUBE family members revealed that the You protein most closely matched SCUBE2 in mouse (65% identity) and SCUBE2 in human (66% identity). The orthology of these genes was further supported by comparative mapping: the human genes *SCUBE2, LMO1, STK33,* and *ST5* exhibited conserved synteny with orthologous genes in both mouse and zebrafish ([49]; unpublished data).

SCUBE proteins are characterized by a signal peptide and by two types of conserved extracellular domains: EGF and CUB [50]. In all identified SCUBE family members, the N-terminal signal sequence is followed by nine EGF repeats, a spacer region, and a single C-terminal CUB domain [48,51,52,53]. Figure 3A shows an alignment comparing the predicted *you* amino acid sequence with selected SCUBE proteins in mouse and human. Similarity between the You protein and mouse SCUBE2 was particularly high in the CUB domain (89% identity), the C-terminal sequence following the CUB domain (90% identity), and the EGF repeats (74% identity). A spacer region in the center of the amino acid sequence showed lower conservation (47% identity). Examination of this spacer region in the vicinity of the CUB domain revealed a repeated motif of six cysteine residues with characteristic and regular spacing, shown in yellow in Figure 3A. Conservation in amino acid sequence was notably higher in this region (66% identical) than in the remainder of the spacer domain (33% identical). This six-cysteine repeat motif does not match the general structure of EGF repeats. The functional significance of this motif is not presently known, but it does lie within a region of SCUBE1 that is required in cell culture for secretion and cell surface expression [52].

**Figure 3:**
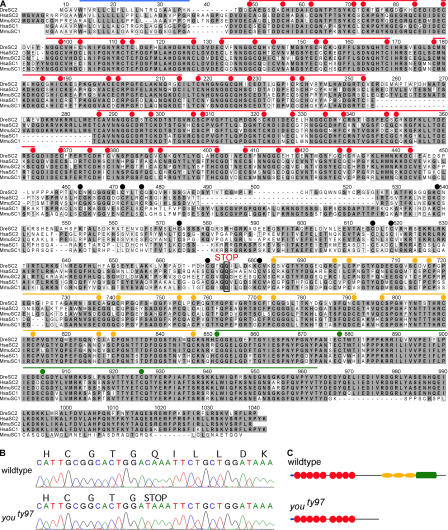
SCUBE Protein Alignment and Truncation of the You Protein In (A) and (C), the signal peptide is labeled in blue, the nine EGF domains are labeled in red, and the CUB domain is labeled in green. (A) Alignment of the predicted You amino acid sequence with SCUBE proteins in mouse and human. Identical amino acids are shaded, and similar amino acids are boxed. Conserved cysteines in these domains and elsewhere in the alignment are indicated by filled circles. A conserved six-cysteine repeat motif N-terminal to the CUB domain is labeled in yellow. The location of the glutamine residue at amino acid 644 in the zebrafish protein, which is changed to a stop codon in *you^ty97^* mutants, is boxed in bold. (B) Sequence traces from homozygous wild-type and *you^ty97^* embryos. In *you^ty97^* mutants, a C to T transition is predicted to change a glutamine codon (CAA) to a stop codon (TAA) and truncate the open reading frame. (C) Model of You protein domain structure. The You protein in *you^ty97^* mutants is predicted to be truncated prior to the six-cysteine repeat motifs, the CUB domain, and the conserved C-terminus.

In *you^ty97^* mutants, a C to T transition alters the coding sequence at residue 644, changing a glutamine codon to a stop codon (Figure 3A and 3B). The predicted mutant protein is truncated immediately prior to the six-cysteine repeat motif, so that these repeats, the CUB domain, and the highly conserved C-terminus are lacking (Figure 3C).

### Morpholino Phenocopy and RNA Rescue of *you*

To confirm that this zebrafish EGF-CUB gene is disrupted in *you* mutants, we performed morpholino oligonucleotide (MO) injection experiments to phenocopy defects seen in *you,* and mRNA injection experiments to rescue the *you* phenotype in mutants (Figure 4). All wild-type embryos injected with a MO targeting the translational start site showed reduced expression of *myod* (*n* = 112) in the adaxial cells (Figure 4B), and an absence of strong Engrailed expression (*n* = 70) in the muscle pioneers (Figure 4D). Embryos injected with a mismatch control MO did not exhibit these defects in either *myod* (*n* = 65) or Engrailed (*n* = 48) expression (Figure 4A and 4C).

**Figure 4:**
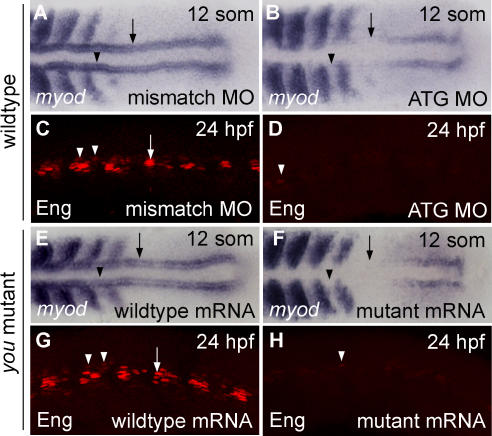
MO-Induced Phenocopy of *you* Defects and Rescue of the *you* Phenotype with mRNA Injection (A, B, E, and F) Dorsal view of the posterior trunk and tail bud of 12-somite embryos. (C, D, G, and H) Lateral views of somites 8–13 in whole-mount embryos at 24 hpf. Anterior is to the left in all images. Injection at the 1–4-cell stage of 420 pg of a MO targeting the translational start site of the *you* mRNA (ATG MO) resulted in decreased adaxial expression of *myod* in the somitic (arrowhead) and presomitic (arrow) mesoderm of wild-type embryos (B). Injection of an equivalent amount of a mismatch control (mismatch MO) did not produce these defects (A). Similarly, wild-type embryos injected with 420 pg of the mismatch MO (C) exhibited strong Engrailed expression in muscle pioneers (arrow) and weaker expression in medial fast fibers (arrowheads). In contrast, Engrailed expression was strongly reduced in wild-type embryos injected with 420 pg of the ATG MO (D), though very weak expression was still observed (arrowhead). Genotypically *you* mutant embryos (E) showed rescued expression of adaxial *myod* in somitic (arrowhead) and presomitic (arrow) mesoderm when injected with 50 pg of synthetic *you* mRNA at the 1–4-cell stage, while mutants injected with 50 pg of a frameshift mutant form of *you* mRNA (F) did not exhibit rescue of adaxial *myod* expression. At 24 hpf, genotypically *you* mutant embryos injected at the 1–4-cell stage with 50 pg of *you* mRNA (G) showed rescue of strong Engrailed expression in the muscle pioneers (arrow) and weaker expression in the medial fast fibers (arrowheads). Mutant embryos injected with 50 pg of the mutant mRNA (H) did not show rescued Engrailed expression, though very weak Engrailed expression (arrowhead) was observed in some cases. Engrailed expression at the MHB was normal in all analyzed embryos (data not shown). Genotypes of embryos shown in (E–H) were determined by PCR after photography.

When injected with 50 pg of synthetic wild-type *you* mRNA, 98.7% (*n* = 665) of embryos from *you/+* intercrosses showed expression of *myod* in adaxial cells at 12 somites (Figure 4E). Genotyping of 571 embryos with wild-type *myod* expression from these intercrosses showed that 137 (24%) were homozygous mutant for *you*. In contrast, 23.6% (*n* = 127) of embryos from *you/+* intercrosses injected with a mutant form of *you* mRNA lacked expression of *myod* in adaxial cells (Figure 4F); 20 of the mutants were genotyped and confirmed to be *you* mutant homozygotes. Similarly, all embryos (*n* = 62) from a *you/+* intercross injected with wild-type mRNA showed strong Engrailed labeling in the muscle pioneers (Figure 4G; genotyping of 32 phenotypically wild-type embryos showed that seven were homozygous *you^ty97^*), whereas 24% (*n* = 33) of embryos injected with control mRNA lacked Engrailed expression (Figure 4H; eight phenotypic mutants were confirmed as homozygous for the *you* mutation). In addition, injection of *you* MOs resulted in loss of *nkx2.2* expression in the trunk and tail of wild-type embryos at 24 hpf, and injection of 50 pg of *you* mRNA was sufficient to rescue *nkx2.2* expression in *you* mutants (data not shown).

### *you* Expression

*you* transcripts appear to be maternally deposited in zebrafish embryos (Figure 5A) and are distributed widely in the embryo through early gastrulation stages (Figure 5B). During late gastrulation, the distribution of *you* transcripts in the embryo began to be restricted, and at bud stage (10 hpf) *you* was expressed in the eye field, in distinct bilateral domains within the developing brain, and in the developing trunk of the embryo in broad paraxial stripes (Figure 5C). During somitogenesis, *you* expression continued to be refined, such that six-somite embryos exhibited expression in the eye field, in stripes in the midbrain and the midbrain–hindbrain boundary (MHB), in a complex pattern in the hindbrain, and in paraxial stripes along the anterior–posterior axis (Figure 5D). At 24 hpf (Figure 5E and 5F), *you* transcripts were localized to the border of the ventral telencephalon and the dorsal diencephalon and to the ventral tectum, and were strongly expressed in the MHB, the hindbrain, and along the length of the embryo in the dorsal spinal cord. In addition, *you* expression was observed in the ventral tail and posterior to the yolk extension at the developing urogenital opening (Figure 5E). In the following 24 h of development, dorsal spinal cord expression continued, and *you* transcripts persisted in a complex and dynamic pattern in the brain. At 48 hpf (Figure 5G and 5H), *you* expression was particularly strong in the cerebellum and in the hindbrain along the rhombic lip.

**Figure 5:**
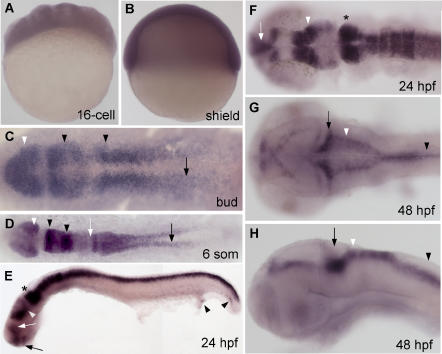
Expression of *you* Examined by In Situ Hybridization (A and B) Maternal *you* transcripts were evident in cleavage-stage embryos (A) (16-cell; 1.5 hpf), and *you* mRNA was widely expressed into the gastrula period (B) (shield stage; 6 hpf). In addition, *you* mRNA was detectable by RT-PCR at 2 hpf, prior to the zebrafish midblastula transition. (C) Toward the end of gastrulation, *you* transcripts began to be restricted, so that at the bud stage (10 hpf), *you* expression was evident in the eye field (white arrowhead), in the developing midbrain and hindbrain (black arrowheads), and in posterior paraxial stripes (arrow). (D) During early somitogenesis (12 hpf), *you* expression was observed in the eye field (white arrowhead), in stripes in the midbrain and the MHB (black arrowheads), in a complex pattern in the hindbrain (white arrow), and in paraxial stripes along the developing trunk and tail bud (black arrow). (E and F) At 24 hpf, *you* transcripts were observed dorsal to the hypothalamus (black arrow), at the boundary between the telencephalon and the diencephalon (white arrow), in the ventral tectum (white arrowhead), in the region of the presumptive cerebellum (asterisk), and dorsally along the length of the spinal cord. Additional expression of *you* at this stage and later was observed in the ventral tail and at the urogenital opening (arrowheads; data not shown). (G and H) At 48 hpf, *you* transcripts were highly expressed in the cerebellum (black arrow), and were also present in the rhombic lip (white arrowhead), and continuing along the length of the anterior–posterior axis in the dorsal spinal cord (black arrowhead; data not shown). Orientation of images: (A) lateral view; (B) lateral view, dorsal to the right; (C, D, F, and G) dorsal views, anterior to the left; (E and H) lateral views, anterior to the left.

### Permissive Role of *you* Upstream of the Hedgehog Cellular Response

To explore the possibility that the *you* gene may induce a gain-of-function phenotype when overexpressed, we injected wild-type embryos with an amount (50 pg) of synthetic *you* mRNA that was sufficient to rescue the phenotypic defects observed in *you* mutants (Figure 6). When compared to embryos injected with a mutant form of *you* mRNA (*myod, n* = 81, Figure 6A; Engrailed, *n* = 25, Figure 6E; *nkx2.2, n* = 17, Figure 6I), overexpression of *you* in wild-type embryos did not result in obvious ectopic expression of *myod* (*n* = 394; Figure 6B)*,* Engrailed (*n* = 25; Figure 6F), or *nkx2.2* (*n* = 17; Figure 6J). This result suggests that *you* functions as a permissive factor in Hedgehog signaling, rather than as a potent activator of the Hedgehog pathway. When 50 pg of synthetic mRNA encoding a potent Hedgehog pathway activator *(shh)* was injected into embryos from a *you/+* intercross, ectopic expression of *myod* (*n* = 53; Figure 6C and 6D), Engrailed (*n* = 78; Figure 6G and 6H), and *nkx2.2* (*n* = 78; Figure 6K and 6L) was induced in all embryos. Genotyping a subset of these embryos indicated that both genotypically wild-type and *you* mutant embryos showed ectopic expression of each of these markers *(myod:* 41 wild-type, 12 *you;* Engrailed: 34 wild-type, 8 *you; nkx2.2:* 63 wild-type, 15 *you).* Because downstream targets of the Hedgehog pathway were rescued or upregulated in *shh-*injected *you* mutants, components of the Hedgehog pathway downstream of *shh* are most likely functional in *you* embryos. These results are consistent with *you* acting upstream of or parallel with *shh* in the Hedgehog pathway.

**Figure 6:**
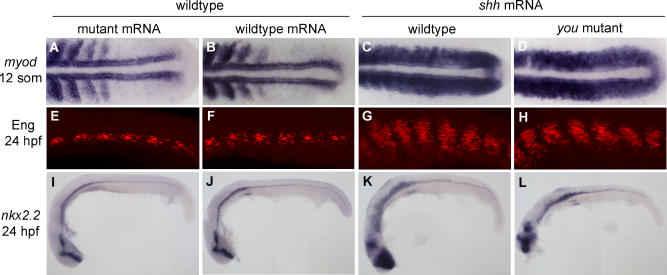
Early Overexpression of *you* in Wild-type Embryos and Rescue of *you* Defects by *shh* mRNA Injection (A–D) Dorsal views of the posterior trunk and tail bud of whole-mount embryos at 12 somites (15 hpf). (E–F) Lateral views of somites 2–7 in 24-hpf embryos. (G–H) Lateral views of somites 8–13 in 24-hpf embryos. (I–L) Lateral views of 24-hpf embryos. Anterior is to the left in all images. When 50 pg of *you* mRNA was injected into wild-type embryos at the 1–4-cell stage, no obvious expansion of *myod* (B), Engrailed (F), or *nkx2.2* (J) expression was observed when compared either with wild-type embryos injected with equivalent amounts of mutant mRNA (A, E, and I) or with uninjected embryos (see Figure 1). Muscle pioneers were counted in a subset of the embryos; there were 4.0 ± 0.8 Engrailed-expressing muscle pioneers per somite in embryos injected with the control mRNA (*n* = 3 embryos, 33 somites) and 4.6 ± 1.1 muscle pioneers per somite in embryos injected with synthetic *you* mRNA (*n* = 8 embryos, 88 somites). Injection of 50 pg of *shh* mRNA into embryos at the 1–4-cell stage resulted in expansion of *myod,* Engrailed, and *nkx2.2* expression in both wild-type (C, G, and K) and *you* mutant (D, H, and L) embryos. s*hh* injection rescued adaxial expression of *myod* (D), muscle pioneer expression of Engrailed (H), and ventral spinal cord expression of *nkx2.2* (L) in genotypically mutant *you* embryos (compare with Figure 1). Genotypes of all embryos were determined by PCR after photography.

Additional evidence that You acts upstream of the Hedgehog response derived from a loss-of-function approach, in which we activated the Hedgehog pathway by knocking down *patched* activity with MOs (Figure 7). Expression of *myod* in adaxial cells was rescued or expanded in all *you* mutant embryos that were injected with MOs targeting *ptc1* (Figure 7E and 7G; *n* = 8 mutants) or a combination of MOs targeting both *ptc1* and *ptc2* (Figure 7I and 7K; *n* = 13 mutants). *you* mutant embryos injected with a *ptc1* mismatch control MO did not exhibit rescued *myod* expression (Figure 7A and 7C; *n* = 8 mutants). In similar experiments, injection of *patched* MOs was sufficient to rescue or expand Engrailed expression in muscle pioneers of *you* mutant embryos (Figure 7B, 7D, 7F, 7H, 7J, and 7L; *ptc1* MO, *n* = 11 mutants; *ptc1* + *ptc2* MO, *n* = 11 mutants).

**Figure 7:**
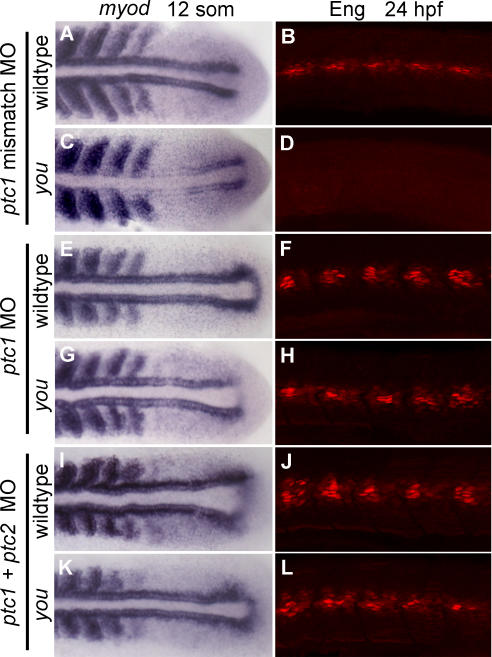
Knockdown of *patched* Function Rescues Slow Muscle Defects in *you* After injection of 420 pg of a mismatch control *ptc1* MO, adaxial expression of *myod* (A) and Engrailed (B) was normal in wild-type embryos, but absent in *you* mutant embryos (C and D). When injected with 420 pg of a MO targeting *ptc1,* however, *myod* expression in mutants (E) was rescued to levels comparable to wild-type embryos (G). Engrailed expression was slightly expanded in both wild-type (F) and mutant (H) embryos injected with 420 pg of *ptc1* MOs. Both adaxial *myod* expression and Engrailed expression was slightly expanded in wild-type (I and J) and *you* mutant embryos (K and L) injected with MOs targeting both *ptc1* and *ptc2* (420 pg each). Embryos assayed for *myod* expression are shown in flat mount at the 12-somite stage, and somites 5–9 of Engrailed-expressing embryos are shown in lateral view at 24 hpf. Anterior is to the left in all panels. Genotypes of all embryos were determined by PCR after photography.

### *you* Acts Non-Autonomously in Muscle Pioneer Differentiation

To determine whether a cell must be wild-type for *you* function to respond to Hedgehog signaling, we created genetic chimeras by transplanting cells from mutant embryos into wild-type hosts (Figure 8). Cells derived from *you* mutant embryos were able to differentiate as muscle pioneers, as defined by characteristic strong Engrailed expression in elongate nuclei of mononucleate cells at the proper position in the somite (Figure 8C–8E; *n* = 13 embryos). Similarly, cells from embryos in which *you* function had been reduced with MOs were able to differentiate as Engrailed-expressing muscle pioneers when introduced into embryos treated with mismatch control MOs (data not shown).

**Figure 8:**
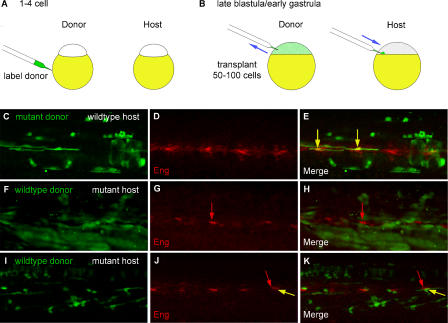
*you* Acts Non-Autonomously in Muscle Pioneers and Is Not Required in Cells Producing Hedgehog Signals (A) Donor embryos were labeled at the 1–4-cell stage with Oregon Green dextran. (B) Cells from donor embryos were transplanted into unlabeled hosts during late blastula and early gastrula stages. (C–E) Images from a chimera made by transplanting cells from labeled mutant donors (green in C and E) into unlabeled wild-type hosts. At 24 hpf, muscle pioneer cells in chimeric embryos were labeled with anti-Engrailed antibody (red in D and E). When transplanted into wild-type embryos, mutant cells were able to differentiate as muscle pioneers, as shown by co-labeling with the anti-Engrailed antibody (E, yellow arrows). (F–K) Images from chimeras made by transplanting cells from labeled wild-type donors (green in F, H, I, and K) into unlabeled mutant hosts. Expression of Engrailed (red in G, H, J, and K) in some mutant muscle pioneers (one marked by red arrows in G, H, J, and K) was rescued in a subset of embryos (see also Table 1). Donor cells in the embryo shown in (F–H) contributed solely to muscle and to non-floor-plate identities within the neural tube. Moreover, in a subset of chimeras, cells derived from wild-type donors differentiated as muscle pioneer cells (yellow arrows in J and K), simultaneously showing both the characteristic strong nuclear Engrailed expression and the typical flattened and mononucleate morphology of this cell type. The somite labeled with the arrows in J and K contains two muscle pioneers, one derived from the wild-type donor (yellow arrow) and another derived from the mutant host (red arrow). Donor cells in the embryo shown in (I–K) contributed primarily to muscle and to non-floor-plate identities within the neural tube; in addition, a group of seven floor plate cells derived from the wild-type donor was present in the tail of this embryo (not shown).

**Table 1:**
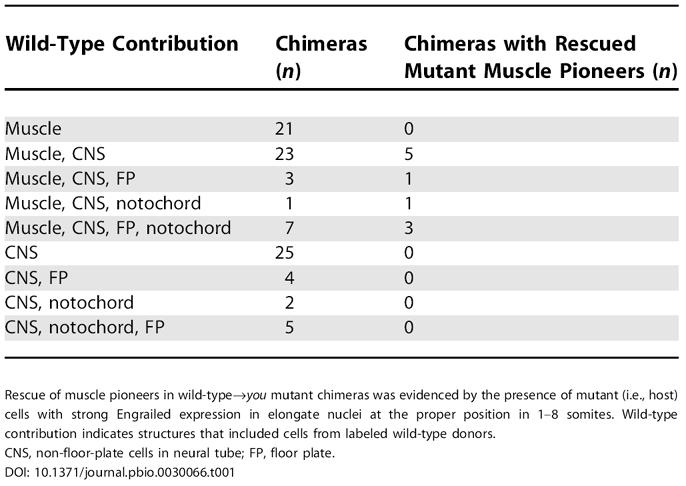
Chimeras with Wild-Type Donor Cells in *you* Mutant Hosts

Rescue of muscle pioneers in wild-type→*you* mutant chimeras was evidenced by the presence of mutant (i.e., host) cells with strong Engrailed expression in elongate nuclei at the proper position in 1–8 somites. Wild-type contribution indicates structures that included cells from labeled wild-type donors

CNS, non-floor-plate cells in neural tube; FP, floor plate

### Muscle Pioneer Differentiation Does Not Require *you* Function in Axial Hedgehog-Producing Cells

To determine which cell types must be wild-type for *you* function in order for target cells to appropriately respond to Hedgehog signals, we transplanted cells derived from wild-type donors into *you* mutant hosts. Of 91 chimeric mutant hosts, ten embryos exhibited rescue of Engrailed expression in genotypically mutant muscle pioneers, as defined by characteristic strong Engrailed expression in elongate nuclei at the proper position of posterior somites, where Engrailed is not normally expressed in *you* mutants (Table 1; Figure 8F–8K). In addition, wild-type cells differentiated as muscle pioneers in two of the ten chimeras with rescued mutant muscle pioneers. In these cases (Figure 8I–8K), the muscle pioneer identity of cells strongly expressing Engrailed was further confirmed by presence of the lineage tracer dye, which showed that these cells had the characteristic flattened and mononucleate morphology of muscle pioneers. In all ten embryos with rescued Engrailed expression, wild-type cells were present in the muscle and in non-floor-plate regions within the neural tube. In five of the chimeric embryos with rescued Engrailed expression, the floor plate and notochord were derived entirely from *you* mutant cells, indicating that *you* function is not required in axial Hedgehog-producing cells. Collectively, the transplantation experiments indicate that *you* function is not required in either the signaling or responding cells, and instead suggest that *you* is essential for the transport or stability of Hedgehog signals.

## Discussion

Using a positional cloning approach, we have shown that the *you* gene encodes a novel EGF-CUB protein essential for Hedgehog signaling in zebrafish. High-resolution mapping indicates that the *you* locus is tightly linked to a zebrafish homolog of mouse *Scube2. you^ty97^* mutants harbor a nonsense lesion that truncates the open reading frame upstream of the CUB domain and other highly conserved sequences. MO-mediated knockdown of the gene phenocopies defects observed in *you* mutants, and injection of wild-type mRNA into mutant embryos rescues the *you* phenotype. Taken together, these experiments provide compelling evidence that the *you* mutation disrupts this EGF-CUB gene.

Biochemical studies of SCUBE family members have shown that they are extracellular, membrane-associated glycoproteins [52,53], but no previous work implicates these proteins in the Hedgehog signaling pathway. Although the Hedgehog pathway has been extensively studied in flies and mammals, several factors have likely obscured the connection between this *Scube* gene and the Hedgehog pathway. Prior to this study, no loss of function analysis had been performed on any *Scube* family gene; there is no known *Scube* gene in the fly, and mouse mutants have not been reported. Also, because overexpression of synthetic *you* mRNA does not significantly hyperactivate Hedgehog signaling, *Scube* gene function in the Hedgehog pathway would not be apparent in gain-of-function screens to identify pathway components.

*you* mutant embryos exhibit phenotypic defects characteristic of reduced Hedgehog signaling in zebrafish, indicating that the *you* gene is a positively acting component of the Hedgehog pathway. Development of slow muscle is disrupted, as shown by lack of adaxial *myod* expression during somitogenesis and the absence of Engrailed-expressing muscle pioneer cells. Moreover, *you* mutants lack *nkx2.2* expression in the ventral spinal cord, showing that specification of ventral neural fates is disrupted in this region of *you* embryos. Additionally, expression of the Hedgehog target gene *ptc1* is reduced in *you* mutants. Analysis of the mutant phenotype, therefore, demonstrates that the *you* gene is essential for Hedgehog signaling in development of slow muscle and ventral spinal cord fates.

Current evidence indicates that EGF-CUB proteins, including You, have extracellular functions. The *you* gene product and other SCUBE proteins contain a signal peptide sequence targeting the protein for secretion, as well as EGF and CUB domains characteristic of extracellular proteins [48,50,51,52,53]. The You homolog SCUBE1 is a glycosylated peripheral membrane protein when expressed in 293T cells, and is also present at low levels in the culture medium [52]. EGF and CUB domains are found together in a small but diverse group of extracellularly acting proteins, including the complement subunits C1s and C1r, the metalloproteinase Tolloid, the sea urchin extracellular matrix protein Fibropellin, the serum glycoprotein Attractin, and the scavenger receptor Cubilin. CUB domains have been implicated in mediating protein–protein interactions and may confer specificity to ligand binding; for example, specific CUB domains in Cubilin have been implicated in facilitating the binding and subsequent endocytosis of specific ligands (reviewed in [54]).

Although *you* is essential for Hedgehog signaling, our analysis indicates that *you* mutant cells are able to produce and respond to Hedgehog signals. *hedgehog* gene expression in the embryonic midline is normal in *you* mutants, indicating that You acts downstream of *hedgehog* gene transcription (see Figure 1). Further evidence that *you* function is not required in cells generating Hedgehog signals derives from the analysis of chimeric embryos: muscle pioneers can differentiate in chimeras in which the notochord and floor plate are formed entirely from mutant cells (see Figure 8; Table 1). Conversely, *you* mutants can respond to Hedgehog pathway activation, mediated by either *shh* overexpression or disruption of *patched,* demonstrating that the defect in the mutants lies upstream of cellular response mechanism (see Figures 6 and 7). In addition, *you* mutant cells can respond to Hedgehog and differentiate as muscle pioneers when transplanted into a wild-type host (see Figure 8), indicating that *you* gene function is not required in cells responding to Hedgehog signals. The analysis of chimeras also demonstrates that the presence of wild-type cells in the paraxial mesoderm and neural plate is sufficient to allow *you* mutant cells to respond to Hedgehog signals produced from *you* mutant notochord and floor plate. Thus, our epistasis and transplantation experiments provide evidence that You functions in the extracellular environment in the field of responding cells, likely acting to transport or stabilize Hedgehog signals. It is possible that You interacts with components of the extracellular matrix, some of which are known to regulate the action of Hedgehog signals in other systems [11,12,55,56].

Another possible model is that You activates the Hedgehog pathway indirectly, by inhibiting an extracellular pathway antagonist such as Hip1 or Gas1. Our results, however, do not support such a model. Overexpression of high levels of synthetic *you* mRNA (up to ten times the amount required to rescue *you* mutants) in wild-type embryos did not result in obvious phenotypes when assayed by gross morphology or by expression of Hedgehog target genes, including adaxial *myod,* Engrailed in muscle pioneers, or *nkx2.2* in the ventral spinal cord (see Figure 6; data not shown). The finding that overexpression of *you* does not significantly hyperactivate Hedgehog targets argues against simple models in which *you* functions to counteract an endogenous repressor of the Hedgehog pathway.

An interesting aspect of the *you* mutant phenotype is that it encompasses only a subset of defects seen in other zebrafish Hedgehog pathway mutants. Whereas mutants for *syu/shh, yot/gli2, smu/smoh,* and *con/disp1* have prominent midline abnormalities in the head, such as ipsilateral retinotectal projections, reduction of anterior pituitary, and defects in medial neurocranial cartilage, these phenotypes are not evident in *you* mutants [26,32,33,37,43,57,58,59]. Also, development of pectoral fins, which is disrupted in *syu, con,* and *smu* [26,60], appears normal in *you* embryos. *you* mutants, therefore, show characteristic Hedgehog signaling defects in slow muscle specification, patterning of ventral spinal cord, and the development of the dorsal aorta, but *you* is apparently not required for Hedgehog signaling in some other regions of the zebrafish embryo. Because the primary cell types disrupted in *you* mutants all develop in close proximity to the notochord, it is possible that *you* gene function may be required for the transport or stability of Hedgehog signals in the vicinity of the developing notochord but not some other regions. The notochord is a defining feature of chordates, and a notochord-associated function would explain why no *you* counterpart is required for Hedgehog signaling in the fly. It is not clear, however, why Hedgehog signaling near the notochord would require a special extracellular mediator. Another possibility is that maternal *you* function could mask earlier requirements in zygotic *you* mutants; future work with maternal-zygotic *you* mutants is needed to address this possibility. A third explanation of the requirement for *you* in only a subset of Hedgehog-regulated processes is that additional factors with redundant functions may substitute for *you* in other regions of the embryo. Intriguingly, expression of another *Scube* gene in mouse—*Scube1—*is observed in many embryonic tissues known to require Hedgehog signaling for their proper development, including the ventral forebrain, limb bud, somites, and developing gonad [48]. These results suggest that an additional zebrafish *Scube* gene may also play a role in the development of other areas of the embryo where Hedgehog signaling is active. Moreover, interactions between SCUBE proteins may be important for Hedgehog signaling; biochemical analysis suggests that SCUBE1 and SCUBE2 proteins can interact to form both homodimers and heterodimers [52].

In addition to its role in promoting Hedgehog signaling in the developing muscle pioneers, ventral neural tube, and dorsal aorta, the expression pattern of *you* suggests that the gene may act in other cell types and perhaps in other pathways. During gastrulation, when Hedgehog signaling is required for specification of muscle pioneers [28], *you* is widely expressed. In 24-hpf embryos, however, *you* is expressed strongly in specific regions in the forebrain, midbrain, and hindbrain, and dorsally along the length of the spinal cord. Some of these expression domains overlap with regions of Hedgehog signaling, whereas others do not. One region where Hedgehog activity and *you* expression intersect at later embryonic stages is the cerebellum, where Hedgehog signaling plays a well-defined role in the proliferation of granule cell precursors in mammals [61,62]. In the trunk and tail, however, *you* expression in the dorsal spinal cord corresponds neither with known sources of Hedgehog signals nor with cells that require Hedgehog signaling for their proper development. This result suggests that *you* may function in other pathways later in development. Future studies will define the role of *you* in the Hedgehog pathway and address the possibility that *you* and other *Scube* family genes also function in other signaling pathways.

## Materials and Methods

### 

#### Fish strains

Zebrafish embryos were maintained at 28.5 °C and were staged according to [63]. Wild-type embryos were derived from the WIK strain. All phenotypic analysis of *you* mutants was performed with embryos homozygous for the *you^ty97^* allele [22].

#### Genetic mapping

The mapping panel was generated by crossing *you^ty97^*/+ individuals with wild-type fish from the WIK strain. *you^ty97^* heterozygotes in the F1 generation were intercrossed, and mutant and wild-type embryos in the F2 progeny were collected at 3–4 dpf for mapping. Genomic DNA was prepared from these embryos as described [64]. Primer sequences for SSLP markers were obtained from the MGH zebrafish database (http://zebrafish.mgh.harvard.edu). For initial localization of *you,* bulked segregant analysis (reviewed in [65]) was performed on DNA pools from 20 mutant and 20 wild-type embryos. Putative zebrafish orthologs of *hedgehog*-related genes were identified by reciprocal BLAST analysis and localized to the Heat Shock Panel as previously described [47].

#### BAC screening, chromosome walking, and BAC sequencing

The CHORI211 BAC library was screened by PCR to identify positive BAC clones (http://www.rzpd.de). BAC end sequences were obtained from the Sanger Institute database (http://trace.ensembl.org, and PCR primers were designed to amplify regions of these sequences. PCR amplicons were sequenced from homozygous wild-type and mutant embryos to identify nucleotide differences that generated restriction enzyme fragment length polymorphisms. These polymorphisms were tested on the mapping panel, and markers showing tighter linkage with *you* were iteratively screened against the BAC library until a contiguous stretch of genomic sequence with ends that flanked *you* was identified. The BAC zC93A15 was subcloned into pBluescript SK+ (Stratagene, La Jolla, California, United States) following double digest with either Pst I and EcoR I or Xba I and Xho I. Sequences were analyzed on a 3730 DNA Analyzer (Applied Biosystems, Foster City, California, United States). Sequences generated from this BAC were used in iterated searches against the zebrafish whole-genome shotgun assembly to nucleate contigs of genomic sequence. Sequencing primers were designed from these contigs and used to generate additional sequence data for zC93A15.

#### Plasmid constructs

A full-length *you* clone in pBluescript SK− (Stratagene) was isolated from a 15–19-hpf cDNA library (gift of Bruce Appel and Judith Eisen). A cDNA clone harboring a frameshift mutation that is predicted to truncate the *you* protein at amino acid residue 34 was isolated from the same library and was used as a control in overexpression experiments. A modified version of the pCS2+ expression vector was generated by cloning a 41-bp fragment into its EcoR I and Xba I sites. This stuffer fragment abolished the endogenous EcoR I, Stu I, Xho I, and Xba I restriction digest sites of pCS2+, and introduced Xba I, Sac I, Apa I, Pst I, and Xho I recognition sequences in a 5′ to 3′ orientation with respect to the SP6 promoter. Wild-type and mutant *you* clones were subcloned into the Xba I and Xho I sites of this modified pCS2+ vector. In situ probes for *you* were generated by linearizing this vector with Xba I, followed by antisense RNA synthesis with T3 polymerase. Synthetic *you* mRNA for injections was generated by digestion with Not I, followed by transcription using the SP6 mMessage mMachine kit (Ambion, Austin, Texas, United States).

#### In situ hybridization, antibody labeling, and genotyping

Probe synthesis, in situ hybridizations, and immunohistochemistry were performed using standard protocols. Embryos from *you*/+ intercrosses were genotyped after in situ hybridization and antibody labeling as described [66]. Other probes used were zebrafish *myod* [67], *ptc1* [68], *nkx2.2* [44], *ehh* [20], *shh* [69], and monoclonal antibody 4D9 [29]. Genotyping was performed by scoring a polymorphism in AI722938 (primer 1, GTGAAAGCAAAAAGCAAGCA; primer 2, GCACTGCATTATGTTTGTGGA; followed by a Hinf I digest).

#### Microinjections

Embryos were injected through their chorions with 500 pl of solution at the 1–4-cell stage as described [70]. RNA was diluted in 0.2 M KCl with 5 mg/ml Phenol Red prior to injection. MOs were obtained from Gene Tools (Philomath, Oregon, United States). A MO targeted to the *you* translational initiation site (5′- GCCGTACAGTCCAAACAGCTCCCAT-3′) or a 5-bp mismatch control MO (5′-GCCcTAg AGTCg AAACAcCTg CCAT-3′) was diluted in a 1x Danieau's solution with Phenol Red at 5 mg/ml prior to microinjection. Sequences for MOs targeting *ptc1* and *ptc2* were obtained from [28].

#### Transplantations

Cellular transplantations were done according to standard methods [22]. Embryos derived from *you/+* intercrosses were injected at the 1–4-cell stage with a 1% solution of Oregon Green 488 dextran (Molecular Probes, Eugene, Oregon, United States). Approximately 50–100 cells were removed from labeled donors in late blastula and early gastrula periods (4–5.3 hpf) and transplanted near the margin of unlabeled sibling hosts. Labeled donor embryos were allowed to develop until 24 hpf. Genotypes of donor embryos derived from *you*/+ intercrosses were determined by PCR. Genotypes of host embryos were determined by staining with Engrailed antibody. Donor cells in chimeras with wild-type cells transplanted into *you* mutant hosts were obtained either from WIK intercrosses or from genotypically wild-type embryos in *you*/+ intercrosses.

## Supporting Information

### Accession Numbers

The *you* cDNA sequence has been deposited in GenBank (http://www.ncbi.nlm.nih.gov/Genbank/index.html) under accession number AY741664.

The LocusLink (http://www.ncbi.nlm.nih.gov/LocusLink/) accession numbers for the genes and gene products discussed in this paper are Attractin (LocusID 8455), C1r (LocusID 715), C1s (LocusID 716), *con/disp1* (LocusID 378448), Cubilin (LocusID 8029), *ehh* (LocusID 30299), Engrailed (LocusID 30244), Gas1 (LocusID 14451), Hedgehog (LocusID 42737), Hip1 (LocusID 15245), Megalin (LocusID 14725), *myod* (LocusID 30513)*, nkx2.2* (LocusID 30697), Patched (LocusID 35851), *prox1* (LocusID 30679), *ptc1* (LocusID 30181), *ptc2* (LocusID 30189), sea urchin Fibropellin (LocusID 373313), *smu/smoh* (LocusID 30225), *syu/ssh* (LocusID 30269), Tolloid (LocusID 42945), *tout velu* (LocusID 36614), *twhh* (LocusID 30444), *ubo/prdm1* (LocusID 323473), *yot/gli2* (LocusID 30154), human *LMO1* (LocusID 4004)*,* human SCUBE1 (LocusID 80274), human SCUBE2 (LocusID 57758), human *ST5* (LocusID 6764), human *STK33* (LocusID 65975)*,* mouse SCUBE1 (LocusID 64706), and mouse SCUBE2 (LocusID 56788).

## Abbreviations

BAC: bacterial artificial chromosome
*con*: *chameleon*
hpf: hours post fertilization
MHB: midbrain-hindbrain boundary
MO: morpholino oligonucleotide
SSLP: simple sequence length polymorphism
